# Early development and school readiness in very preterm children

**DOI:** 10.3389/fped.2026.1818425

**Published:** 2026-04-23

**Authors:** Kamini Raghuram, Nicole Bando, Magdalena Janus, Seungwoo Lee, Ashley Gaskin, Amanda Offord, Caroline Reid-Westoby, Rachel Ault, M. Florencia Ricci, Marie-Noelle Simard, Prakesh S. Shah

**Affiliations:** 1 Department of Paediatrics, Mount Sinai Hospital, Toronto, ON, Canada; 2 Department of Pediatrics, St. Michael’s Hospital, Unity Health Toronto, Toronto, ON, Canada; 3 Offord Centre for Child Studies, Department of Psychiatry and Behavioural Neurosciences, McMaster University, Hamilton, ON, Canada; 4 Maternal-Infant Care Research Centre, Mount Sinai Hospital, Toronto, ON, Canada; 5 Ontario Institute for Studies in Education, University of Toronto, Toronto, ON, Canada; 6 Department of Pediatrics and Child Health, University of Manitoba, Winnipeg, MB, Canada; 7 School of Rehabilitation, Faculty of Medicine, Université de Montréal, Montréal, QC, Canada

**Keywords:** early development instrument, neonatal intensive care, neurodevelopment, preterm, school readiness

## Abstract

**Purpose:**

Children born very preterm often experience difficulties in academic performance at school entry, which may persist and affect long-term outcomes. Few studies have examined school readiness among children born very preterm, and routine follow-up to school age is uncommon. This study aims to describe school readiness in very preterm children and assess associations between neurodevelopmental outcomes at 18–24 months corrected age and later school readiness.

**Methods:**

This study included 112 children born <29 weeks’ gestation in Ontario, Canada (birth years 2009 and 2012), who completed the assessments of the Bayley Scales of Infant and Toddler Development, 3rd edition (BSID-III) at 18–24 months corrected age. Deterministic linkage connected BSID-III results with the Early Development Instrument (EDI), a kindergarten teacher-completed measure of school readiness. Vulnerability—defined as scores below the 10th percentile—was compared between the very preterm cohort and their Ontario peers and between children with and without neurodevelopmental impairment at 18–24 months.

**Results:**

Children born very preterm were more than twice as likely to be vulnerable in two or more EDI domains compared with their Ontario peers (34% vs. 14%). Those with neurodevelopmental impairment at 18–24 months showed higher vulnerability across all domains. Notably, 35% of children without neurodevelopmental impairment still demonstrated vulnerability at school entry. Lower BSID-III language scores were associated with vulnerability in physical health and wellbeing, language and cognitive development, and communication skills and general knowledge.

**Conclusion:**

Very preterm children, especially those with early neurodevelopmental impairment, were at increased risk for vulnerability across all areas of school readiness. Attention to early language development may help improve later school readiness outcomes.

## Introduction

School readiness is a multifaceted, interdependent, and dynamic concept that includes a child’s physical health, cognitive and early academic abilities, ability to communicate effectively, social reciprocity, behavior, and emotional regulation (1). This comprehensive, function-based definition has been affirmed by the UNICEF school readiness framework and is an important outcome for caregivers and policymakers alike (2). It is important to recognize that according to this definition, while academic abilities form part of a child’s readiness for school, other developmental aspects play an equally important role and should be measured as outcomes.

The literature has clearly documented that children born very preterm (VPT) face challenges in academic performance at school entry, which persist throughout the years and ultimately impact employment and social relationships in adulthood (3–10). Data suggest that over a third of children born VPT require special education and 15% repeat a grade in school (11, 12). Unfortunately, in Canada, while routine developmental follow-up is performed at 18–36 months and collected in a large national database, no systematic data are available beyond this age. A few small Canadian cohort studies have reported on academic performance at entry to school (13, 14). For example, Synnes et al. (14) detected higher rates of cognitive delays at school entry not detected in toddlerhood. The major limitations of these studies are that (1) they are limited in scope and size and (2) do not address the full spectrum of school readiness, including physical health and emotional regulation, which are predictors of school success (15) and determine the degree of support needed in school (16, 17).

The use of a validated school readiness measure incorporating aspects of development such as physical health and emotional regulation in the VPT population would help address this gap. The Early Development Instrument (EDI) (18, 19) is one such tool that is currently used in Canada and internationally at the population level to identify groups of children at risk of academic underperformance. Data from EDI assessments are also collected systematically at the provincial level. Recently, a study in Manitoba using the EDI as an outcome has shown that only 65% of preterm children are ready for school compared with 85% of children born at term (20). This is at variance with outcomes at the toddler stage suggesting that fewer than 10% of preterm children have significant delays (21). A few studies have related neurodevelopmental outcomes in toddlerhood with later cognitive ability and academic performance (11, 22), but no studies have described the relationship between the assessments done at 18–36 months and a holistic school readiness measure like the EDI in Canada.

Accordingly, together with our parent partner, our research question focused on understanding the school readiness profiles of children born very preterm and determining how early developmental assessments currently undertaken in neonatal follow-up correlate with school readiness in this population. Our study objectives were therefore to describe the school readiness profile of Ontario children born VPT and to explore how the 18–36 month developmental assessment was associated with later school readiness.

## Method

### Study participants and design

This study was conducted with children born in the years 2009 and 2012 in Ontario, Canada. These years corresponded to the years 2015 and 2018, when these children were in kindergarten and when the EDI was collected in Ontario as part of the Ministry of Education’s routine developmental assessments of kindergarten children.

Participants for this study included preterm neonates born <29 weeks’ gestational age (GA) who attended a neonatal neurodevelopmental follow-up (NNFU) clinic that contributes data to the Canadian Neonatal Follow-up Network (CNFUN). In Ontario, children born VPT are followed up at a NNFU clinic and standardized testing is performed between 18 and 36 months corrected age (CA). Children who did not complete at least one Bayley Scales of Infant and Toddler Development, 3rd edition (BSID-III) (23) assessment or who did not have EDI data available in either 2015 or 2018, had major congenital or chromosomal anomalies, or who died prior to follow-up were excluded from this study. According to the Canadian Pediatric Society, corrected age is used for children until their chronologic age is 3 years, while chronologic age is used thereafter (24). Table 1 gives the details of the participants included in this study; a total of 112 children were included in this study.

**Table 1:** Characteristics of the study sample.

	Typically developing (*N* = 62)	NDI (*N* = 50)	*p*-Value
Maternal characteristics			
Age, median (IQR), year	32.5 (29.0, 37.0)	33.0 (30.0, 39.0)	0.52
Parity, median (IQR)	1 (0, 1)	1 (0, 2)	0.08
Primary caregiver education, college and above	52 (89.7)	36 (92.3)	0.74
Neonatal characteristics			
Birth weight, mean (SD), and g	1001 (264)	972 (216)	0.54
Birth gestational age, median (IQR), and week	27.0 (26.0, 28.0)	26.5 (25.0, 28.0)	0.50
Male sex	32 (51.6)	27 (54.0)	0.80
Multiple gestation	10 (16.1)	3 (6.0)	0.10
SNAP-II, median (IQR)	9.0 (5.0, 14.0)	9.5 (8.0, 14.0)	0.44
5 min APGAR score and median (IQR)	7 (6, 9)	7 (5, 8)	0.12
Grade III/IV intraventricular hemorrhage or periventricular leukomalacia	4 (6.6)	7 (14.3)	0.21
Bronchopulmonary dysplasia	26 (42.6)	20 (40.0)	0.78
Late-onset sepsis	16 (25.8)	10 (20.0)	0.47
Follow-up characteristics			
Chronologic age at follow-up, median (IQR) (range), and months	21.36 (21.00, 22.03) [19.75, 33.69]	21.64 (21.26, 22.82) [19.92, 33.20]	0.08
Corrected age at follow-up, median (IQR) (range), and months	18.5 (18.1, 19.1) [17, 30.39]	18.5 (18.2, 19.8) [17.33, 30.56]	0.33

Data are presented as *n* (%) unless otherwise specified. Data are compared with typical development by using a Chi-square test or Fisher’s exact test as appropriate for categorical variables and Student’s *t*-test or the Wilcoxon rank-sum test for continuous variables.

NDI, neurodevelopmental impairment; SNAP-II, score for neonatal acute physiology-II.

The Canadian Neonatal Network (CNN) and CNFUN maintain a national standardized database (hereafter referred to as the CNN database) of neonatal diagnoses and treatments and neurodevelopmental outcomes for infants of <29 weeks’ GA admitted to level III Neonatal Intensive Care Units (NICUs) in Canada. Three identifiers (birth date, postal code, and sex) were used to conduct deterministic linkage of the CNN database with the EDI database, housed at the Offord Centre for Child Studies, McMaster University. During this process, if duplicates were found, they were excluded from the study. Therefore, all same-sex multiples were excluded as there was no possibility to use other identifiers to separate their information.

Children’s baseline characteristics, including maternal and neonatal characteristics, were obtained from the CNN database. Neurodevelopmental impairment (NDI) was determined based on previously used CNFUN criteria. These include the diagnosis of cerebral palsy (25), assessment of the child’s motor function using the Gross Motor Function Classification Scale (GMFCS), standardized developmental testing using BSID-III for motor, cognitive, and language skills, and a combination of parent report and available medical records for assessment of neurosensory (i.e., hearing and vision) function. BSID-III is a standardized assessment of cognitive, language, and motor subtests, each with a mean score of 100 and a standard deviation (SD) of 15. A child performing at <1 SD below the mean is considered to have a delay in that particular area. This is a previously established cutoff used extensively in studies published using CNN and CNFUN data (26) and supported by literature describing overestimation of development with BSID-III compared with the Bayley Scales of Infant Development, 2nd edition (BSID-II) (27). If a child were delayed in any area of BSID-III, had a diagnosis of cerebral palsy, or a neurosensory impairment, they were characterized as having NDI. Children without any of these delays detected by 18–36 months were considered to be typically developing.

School readiness was assessed using the EDI, a 103-item, teacher-completed questionnaire that assesses a child’s ability to meet age-appropriate developmental expectations in five developmental domains—physical health and wellbeing, social competence, emotional maturity, language and cognitive development, and communication skills and general knowledge (19). The assessment is completed during the child’s senior kindergarten year, which is typically when the child is 5–6 years old and tends to be completed toward the end of the academic year. The mean EDI scores for each of the domains were collected for all children who were matched from the CNN database to the EDI database. Mean scores for each developmental domain range from 0 to 10, where a higher score indicates greater ability in a given area. Mean domain scores for children born VPT were compared with their Ontario peers, data that are available publicly for the same testing years (i.e., 2015 and 2018). A child is considered “vulnerable” in a domain if their score falls below the 10th percentile, based on a normative population. The EDI has been validated for the general population in Canada, as well as in children with special needs (18, 28). One large Canadian study has also used this specific cutoff for children born very and extremely preterm (20). Supplementary Table A1 shows samples of questions included in the EDI.

### Statistical analysis

Descriptive statistics were conducted to summarize the maternal, neonatal, and follow-up characteristics of the study sample and to compare children born VPT who were typically developing with those who had NDI. Continuous variables were displayed as means and standard deviations or medians and interquartile ranges for normally and non-normally distributed data, respectively, while categorical variables were displayed as counts and percentages. Continuous variables were assessed for normality by evaluating kurtosis, skewness, and the Kolmogorov–Smirnov test. Student’s *t*-test/Satterthwaite *t*-test and Wilcoxon rank-sum tests were used for normally and non-normally distributed variables, respectively, to obtain the *p*-values as appropriate. Chi-square and Fisher’s exact tests were used as appropriate for categorical variables. Missing data were assumed to be missing completely at random, and a comparison of baseline characteristics is available in Supplementary Table A2.

EDI results were compared between Ontario peers and all children born VPT. Comparisons were also made between Ontario peers and VPT children with and without NDI at 18–36 months. For each comparison, EDI mean scores and vulnerability were calculated for each domain, and vulnerability in ≥1 domains, ≥2 domains, and all domains were compared. Statistical adjustment for confounding was performed using multivariable logistic regression models accounting for variables determined *a priori* to be associated independently with neurodevelopment (i.e., gestational age, sex, small-for-gestational age, and caregiver education). To account for multiples included in the study, generalized estimating equations models were used. A *post-hoc* power calculation suggested that this sample size was adequate to detect a 25% difference in EDI vulnerability in at least one domain between children with NDI and those without NDI.

All analyses were conducted using SAS 9.4 (SAS Institute Inc., Cary, NC, USA).

## Results

Of 503 VPT children who attended follow-up at 18–36 months CA, 112 children were successfully linked to the EDI database and included in the analysis of outcomes (22% match rate). Sixty-two children (55%) were developing typically, and 50 (45%) were identified as having NDI at 18–36 months (Table 1). There were no baseline differences between these groups. Supplementary Table A3 shows the developmental domain(s) involved in determining NDI. Of note, 80% of children who were characterized as having NDI had cognitive composite scores < 85.

Overall, this study found 46% of children born VPT were considered vulnerable in ≥1 developmental domains at school entry and 33% were vulnerable in ≥2 domains. In comparison, 29.5% of Ontario peers were vulnerable in ≥1 developmental domains, while only 14.2% were vulnerable in ≥2 domains (Table 2**)**. Compared with their Ontario peers, children born VPT had lower mean scores in all domains of the EDI and many of them were vulnerable in all EDI domains. A greater percentage of children without NDI at 18–36 months were vulnerable in social competence and language and cognitive development and in ≥2 domains compared with their Ontario peers. VPT children without NDI at 18–36 months generally functioned similarly to their Ontario peers, although 35% were still found to be vulnerable in ≥1 developmental domains and 27% in ≥2 domains.

**Table 2:** Comparison of EDI data of very preterm children and their Ontario peers.

	Ontario peers (*N* = 249 770)	Preterm (*N* = 112)	*p*-Value	Typically developing preterm (*N* = 62)	*p*-Value	Preterm with NDI (*N* = 50)	*p*-Value
Age at assessment							
Age at EDI assessment, mean (SD), and months	68.8 (3.5)	67.7 (3.0)	**0**.**0003**	67.8 (3.0)	**0**.**008**	67.7 (3.1)	**0**.**01**
EDI domain scores and mean (SD)							
Physical health and wellbeing	8.80 (1.33)	8.04 (1.87)	**<0**.**0001**	8.62 (1.36)	0.30	7.30 (2.16)	**<0**.**0001**
Social competence	8.32 (1.85)	7.50 (2.28)	**0**.**0001**	7.79 (2.25)	0.06	7.14 (2.28)	**0**.**0003**
Emotional maturity	8.04 (1.58)	7.45 (1.81)	**0**.**0006**	7.60 (1.80)	0.054	7.27 (1.82)	**0**.**003**
Language and cognitive development	8.83 (1.59)	8.07 (2.37)	**0**.**0007**	8.61 (1.64)	0.29	7.42 (2.91)	**0**.**0006**
Communication and general knowledge	7.96 (2.43)	6.85 (2.83)	**<0**.**0001**	7.51 (2.41)	0.14	6.04 (3.10)	**<0**.**0001**
EDI vulnerability (<10th percentile), *n* (%)							
Physical health and wellbeing	40 477 (16.2)	34 (30.4)	**<0**.**0001**	11 (17.7)	0.88	23 (46.0)	**<0**.**0001**
Social competence	25 710 (10.3)	25 (22.5)	**<0**.**0001**	14 (23.0)	**0**.**002**	11 (22.0)	**0**.**01**
Emotional maturity	29 430 (11.8)	25 (22.5)	**0**.**0008**	12 (19.7)	0.09	13 (26.0)	**0**.**004**
Language and cognitive development	17 779 (7.1)	19 (17.1)	**<0**.**0001**	6 (9.8)	**0**.**0001**	13 (26.0)	**<0**.**0001**
Communication and general knowledge	25 189 (10.1)	24 (21.6)	**0**.**0001**	8 (13.1)	0.57	16 (32.0)	**<0**.**0001**
EDI vulnerability ≥1 domain, *n* (%)	73 671 (29.5)	52 (46.4)	**0**.**0001**	22 (35.5)	0.37	30 (60.0)	**<0**.**0001**
EDI vulnerability ≥2 domains, *n* (%)	35 389 (14.2)	38 (33.9)	**<0**.**0001**	17 (27.4)	**0**.**005**	21 (42.0)	**<0**.**0001**
EDI vulnerability in all domains, *n* (%)	3061 (1.2)	7 (6.3)	**0**.**0005**	2 (3.2)	0.18	5 (10.0)	**0**.**0004**

Data are compared with Ontario peers by using a Chi-square test or Fisher’s exact test for categorical variables and the Satterthwaite *t*-test for continuous variables.

EDI, early development instrument; NDI, neurodevelopmental impairment. Bolded: *p* < 0.05.

Table 3 shows the mean EDI domain scores and EDI vulnerability VPT children with and without NDI and with and without adjustment for confounding. Accounting for gestational age, sex, small-for-gestational age, and caregiver education did not impact the results. Significant differences were noted in mean EDI scores and EDI vulnerability in terms of physical health and wellbeing, language and cognitive development, and communication and general knowledge and in EDI vulnerability in ≥1 domain.

**Table 3:** Comparison of EDI data of very preterm children with typical development and with NDI.

	Typically developing (*N* = 62)	NDI (*N* = 50)	Unadjusted OR (95% CI)	Adjusted OR (95% CI)
Age at assessment				
Age at EDI assessment, mean (SD), months	67.78 (2.99)	67.69 (3.13)	0.91 (0.29, 2.81)	0.80 (0.24, 2.60)
EDI domain scores and mean (SD)				
Physical health and wellbeing	8.62 (1.36)	7.30 (2.16)	**0.27** (**0.14, 0.51)**	**0.25** (**0.13, 0.50)**
Social competence	7.79 (2.25)	7.14 (2.28)	0.52 (0.23, 1.21)	0.56 (0.23, 1.34)
Emotional maturity	7.60 (1.80)	7.27 (1.82)	0.72 (0.37, 1.41)	0.73 (0.35, 1.51)
Language and cognitive development	8.61 (1.64)	7.42 (2.91)	**0.30** (**0.13, 0.71)**	**0.30** (**0.13, 0.72)**
Communication and general knowledge	7.51 (2.41)	6.04 (3.10)	**0.23** (**0.08, 0.63)**	**0.30** (**0.10, 0.91)**
EDI vulnerability (<10th percentile), *n* (%)				
Physical health and wellbeing	11 (17.7)	23 (46.0)	**3.95** (**1.68, 9.30)**	**4.70** (**1.75, 12.59)**
Social competence	14 (23.0)	11 (22.0)	0.95 (0.39, 2.32)	0.99 (0.36, 2.69)
Emotional maturity	12 (19.7)	13 (26.0)	1.43 (0.59, 3.50)	1.43 (0.53, 3.86)
Language and cognitive development	6 (9.8)	13 (26.0)	**3.22** (**1.12, 9.23)**	**3.74** (**1.19, 11.77)**
Communication and general knowledge	8 (13.1)	16 (32.0)	**3.12** (**1.20, 8.08)**	**2.94** (**1.02, 8.50)**
EDI vulnerability ≥1 domain, *n* (%)	22 (35.5)	30 (60.0)	**2.73** (**1.26, 5.88)**	**2.97** (**1.21, 7.29)**
EDI vulnerability ≥2 domains, *n* (%)	17 (27.4)	21 (42.0)	1.92 (0.87, 4.23)	1.88 (0.76, 4.66)
EDI vulnerability in all domains, *n* (%)	2 (3.2)	5 (10.0)	3.33 (0.62, 17.97)	3.93 (0.62, 24.85)

Data are analyzed using unadjusted linear and logistic regression models and multiple linear and logistic regression models adjusted for gestational age, sex, small-for-gestational age, and caregiver education. Normal distribution with the identity link is used for continuous variables. Bolded: *p* < 0.05.

The BSID-III language subtest was associated with physical health and wellbeing, language and cognitive development, communication skills and general knowledge, and vulnerability in ≥1 domain and ≥2 domains (Table 4**)**. The BSID-III cognitive subtest was associated only with physical health and wellbeing. The BSID-III motor subtest was not associated with vulnerability in any of the EDI domains. None of the BSID-III subtests were associated with social competence or emotional maturity.

**Table 4:** Association between BSID-III cognitive and language scores and EDI vulnerability in very preterm children.

EDI vulnerability (<10th percentile)	BSID-III cognitive score (*N* = 109)				BSID-III language score (*N* = 109)			
	<85, *n*/*N* (%)	≥85, *n*/*N* (%)	Unadjusted OR (95% CI)	Adjusted OR (95% CI)	<85, *n*/*N* (%)	≥85, *n*/*N* (%)	Unadjusted OR (95% CI)	Adjusted OR (95% CI)
Physical health and wellbeing	13/23 (56.5)	21/86 (24.4)	**4.02** (**1.54, 10.51)**	**4.76** (**1.46, 15.49)**	19/39 (48.7)	15/70 (21.4)	**3.48** (**1.49, 8.14)**	**4.07** (**1.52, 10.88)**
Social competence	6/23 (26.1)	19/85 (22.4)	1.23 (0.42, 3.54)	1.07 (0.30, 3.90)	10/39 (25.6)	15/69 (21.7)	1.24 (0.50, 3.11)	1.50 (0.54, 4.16)
Emotional maturity	4/23 (17.4)	21/85 (24.7)	0.64 (0.20, 2.10)	0.58 (0.15, 2.32)	10/39 (25.6)	15/69 (21.7)	1.24 (0.50, 3.11)	1.34 (0.48, 3.72)
Language and cognitive development	6/23 (26.1)	13/85 (15.3)	1.95 (0.65, 5.89)	1.89 (0.49, 7.22)	12/39 (30.8)	7/69 (10.1)	**3.94** (**1.40, 11.09)**	**6.22** (**1.86, 20.79)**
Communication and general knowledge	8/23 (34.8)	16/85 (18.8)	2.30 (0.83, 6.35)	2.21 (0.63, 7.71)	15/39 (38.5)	8/69 (11.6)	**4.77** (**1.79, 12.69)**	**5.76** (**1.86, 17.84)**
≥1 domain	15/23 (65.2)	37/86 (43.0)	2.48 (0.95, 6.47)	3.32 (0.99, 11.11)	25/39 (64.1)	26/70 (37.1)	**3.02** (**1.34, 6.82)**	**3.20** (**1.24, 8.29)**
≥2 domains	11/23 (47.8)	27/86 (31.4)	2.00 (0.79, 5.11)	1.80 (0.57, 5.72)	19/39 (48.7)	19/70 (27.1)	**2.55** (**1.12, 5.79)**	**2.62** (**1.02, 6.73)**
All domains	2/23 (8.7)	5/86 (5.8)	1.54 (0.28, 8.52)	1.00 (0.10, 9.99)	4/39 (10.3)	3/70 (4.3)	2.55 (0.54, 12.05)	5.14 (0.82, 32.39)

Data are analyzed using unadjusted logistic regression models and multiple logistic regression models adjusted for gestational age, sex, small-for-gestational age, and caregiver education. Bolded: *p* < 0.05.

## Discussion

The results from this study indicate that children born VPT are less ready at school entry, and although the subgroup of children with NDI at 18–36 months CA may be particularly vulnerable at school entry, even children without NDI require special attention. Two other Canadian studies have reported on school readiness of very preterm children as measured with the EDI (20, 29). One population-based cohort (*n* = 89, <28 weeks GA; *n* = 796, 28–33 weeks) in the province of Manitoba found similar rates of vulnerability in EDI domains as the present study (20). Also reported from Manitoba was a higher prevalence of vulnerability in multiple domains among children born <28 weeks GA compared with term-born children. We too found a significant difference between very preterm children and their Ontario peers with EDI vulnerability in ≥2 domains (34% vs 14%); this percentage reached 42% in children with NDI at 18–36 months CA. In 77 very low birth weight children (median GA: 27 weeks) from Ontario’s tertiary neonatal units in Toronto, proportions of vulnerability in physical health and wellbeing, communication skills and general knowledge, and vulnerability in ≥2 domains were significantly higher than those in their Ontario peers, including 26% vulnerability in ≥2 domains (29). The proportion of vulnerability for each EDI domain was lower than that of our results, although differences in neonatal morbidity prevalence were noted between the study samples.

Given that 80% of children in this study characterized as having NDI had cognitive scores on BSID-III < 85, understanding and monitoring cognition in this population of VPT children is a priority. Other studies have shown that while children born VPT on average perform within the typical age-expected range, a higher proportion (OR range 3.4–6 compared with term children) of children born VPT have lower scores on standardized assessments of cognitive function, and the scores differ from those of full-term infants by approximately one SD (30–33). Importantly, even in children without demonstrable delays at 2 years, 15% require some degree of special education, and VPT children were twice as likely to need an individualized education plan and were 1.5 times more likely to repeat a grade in school (12, 34, 35). Therefore, characterizing and understanding cognitive function would help us better understand the challenges they face in academic performance at school entry (3) and also help us continue to support them throughout the lifespan (8, 9, 12).

While cognition underlies some of the challenges in school readiness, social and emotional regulation is an equally important aspect of overall functioning and school readiness in VPT children. However, social and emotional regulation frequently becomes a concern for parents who have limited resources to support it (36, 37). Our study uniquely evaluates these aspects of school functioning and shows differences in social competence and emotional regulation in preterm children compared with the general population, which may impact classroom functioning (38). Routine screening for these aspects of development, together with increased teacher awareness of these unique developmental issues related to prematurity (39), would allow for the implementation of specific classroom strategies that will ultimately optimize academic performance.

Studies examining the relationship between early developmental assessments (e.g., the BSID-III) and school readiness are sparse. One study that examined the relationship between BSID-II results and longer-term school-aged outcomes reported that 50% of children with BSID-II scores <2 SD from the mean were not ready for school, and that while only 23% of children were considered delayed at the age of 2 years, 32% at the age of 5 years required special education at school (11). In this study, the predictive value of a score <2 SDs from the mean on the BSID-II was only 50% predictive of the lack of school readiness at 5 years. Previous literature has shown that BSID-III overestimates development in children at the age of 2 years (27), and therefore, delays of <1 SD below the mean are considered equivalent to BSID-II delays of <2 SD below the mean. Therefore, our study, similar to Patrianakos et al, suggests that a higher proportion of children at school-age struggle with school readiness, and that these children are missed if we rely only on the 2-year visit to describe their developmental profile or if we fail to continue to follow up these children beyond this time point. In addition, while a BSID-III assessment done at 18–36 months may capture some aspects of school readiness, it may not be relevant for behavioral, social, or emotional regulation skills necessary for school readiness.

This study demonstrates that the language subtest of BSID-III was associated with children’s communication, language, and cognitive skills at school age. Numerous studies have demonstrated the importance of early language exposure in developing cognitive abilities and improving academic performance (40). In addition, the BSID-III language and cognitive subtests were associated with physical health and wellbeing. The physical health and wellbeing (19) domain encompasses questions around physical readiness for the school day (e.g., if the child was too tired or sick to do schoolwork), their level of independence (e.g., use of the washroom), and their ability to perform gross and fine motor tasks (e.g., climbing stairs and using a pen or crayon). Many of these tasks rely on cognitive function and ability to communicate, hence the emerging association. Thus, this may indicate that strategies aimed specifically at supporting language development, especially for children with delayed language on the BSID-III assessment at 18–36 months, are particularly important in promoting school readiness in the VPT population.

This study has several strengths, including the use of two large, validated population-based databases to assess educational outcomes. In addition, the study team included a parent representative with a background in education to advise the research question, methodology, reporting, and knowledge translation. Finally, the use of a teacher rating of school readiness provided valuable, naturalistic information on the child’s developmental status in different ability domains rather than relying on one standardized direct assessment done in a clinic environment. The main limitation of this study related to linkage. For instance, children who moved location before school entry and same-sex multiples could not be matched. As serious neonatal morbidity prevalence was lower in our cohort than that reported from the Canadian Neonatal Network, we hypothesize that our results underestimate the true difficulties of the VPT population at school age. Together with the growing evidence in the literature on concerns about inattention and social communication difficulties in children born preterm (41), it is imperative that system-level linkage between healthcare and school be available. This will allow other provinces to understand the longer-term outcomes of their VPT population within their education system (20) and also allow parents, healthcare providers, policymakers, and educators to better understand the needs of this population and create comprehensive support systems prior to and after entry into school (19).

Another limitation of this study was the ability to correct for confounding in the comparison between children born VPT and Ontario peers (42). However, adjustment for socioeconomic confounding was performed for children with NDI and those developing typically, and it did not appear to change the results in EDI vulnerability, indicating that prematurity itself carries with it a predisposition to delayed school readiness that should be recognized and addressed. We also recognize that the definition of NDI varies worldwide, and many areas use a cutoff of <70 indicating a 2-SD difference from the mean as significant. The use of a cutoff of <85 may underestimate children’s abilities. However, despite this higher cutoff, significant differences in the EDI were noted in this study, indicating that development continues to evolve in the period before entry into school and continued follow-up is imperative.

In summary, VPT children are vulnerable to delays in school readiness, even if their assessments at 18–36 months show typical development. It is imperative to follow up children born VPT beyond 18–36 months systematically and develop specific interventions to target the preschool years to aid these children in optimizing school functioning.

## Data Availability

The datasets presented in this article are not readily available because the data were analyzed within the Canadian Neonatal Network, and REB approval does not permit sharing them outside the CNN and/or CNFUN. Further enquiries can be directed to the corresponding author.
