# Supplementary material for: Early development and school readiness in very preterm children
Source: Front Pediatr. 2026 Apr 23;14:1818425. doi: 10.3389/fped.2026.1818425 (PMC13149230; doi:10.3389/fped.2026.1818425)
Supplement: Supplementary file 1 [file Supplementaryfile1.docx]

**Supplementary material**

**Table A1: Sample question in Early Development Instrument (EDI)**

| EDI Domains | Subdomains | Example items |
| --- | --- | --- |
| Physical Health and Well-being | Physical readiness for school day | arriving to school hungry |
|  | Physical independence | having well-coordinated movements |
|  | Gross and fine motor skills | being able to manipulate objects |
| Social Competence | Overall social competence | ability to get along with other children |
|  | Responsibility and respect | accept responsibility for actions |
|  | Approaches to learning | working independently |
|  | Readiness to explore new things | eager to explore new items |
| Emotional Maturity | Pro-social and helping behaviour | helps other children in distress |
|  | Anxious and fearful behaviour | appears unhappy or sad |
|  | Aggressive behaviour | gets into physical fights |
|  | Hyperactivity and inattention | is restless |
| Language and Cognitive Development | Basic literacy | able to write own name |
|  | Interest in literacy/numeracy and memory | interested in games involving numbers |
|  | Advanced literacy | able to read sentences |
|  | Basic numeracy | able to count to 20 |
| Communication Skills and General Knowledge | (No subdomains) | able to clearly communicate one’s own needs and understand others  shows interest in general knowledge about the world |

**Table A2: Characteristics of very preterm children included and not included in analysis**

|  |  | **EDI data available (N=112)** | **EDI data not available (N=391)** | **p-value** |
| --- | --- | --- | --- | --- |
| **Maternal characteristics** |  |  |  |  |
| Age, mean (SD), y |  | 32.9 (5.7) | 31.4 (5.7) | **<0.0001** |
| Parity, median (IQR) |  | 1 (0, 1) | 0 (0, 1) | 0.10 |
| Primary caregiver education, college and above |  | 88 (91) | 266 (81) | **0.04** |
| **Neonatal characteristics** |  |  |  |  |
| Birth weight mean (SD), g |  | 988 (243) | 933 (220) | 0.08 |
| Birth gestational age, median (IQR), wk |  | 27 (26, 28) | 27 (25, 28) | 0.85 |
| Male sex |  | 59 (53) | 210 (54) | 0.72 |
| Small-for-gestational age |  | 5 (4) | 28 (7) | 0.58 |
| Multiple gestation |  | 13 (12) | 148 (38) | **<0.0001** |
| Severe brain injury^1^ |  | 11 (10) | 46 (12) | **0.045** |
| Bronchopulmonary dysplasia |  | 46 (41) | 151 (39) | 0.07 |
| Necrotizing enterocolitis Stage II/III |  | 3 (3) | 18 (5) | 0.62 |
| Retinopathy of prematurity requiring treatment |  | 6 (6) | 22 (6) | 0.79 |
| Late-onset sepsis |  | 26 (23) | 96 (25) | **0.005** |

Data presented as n (%) unless otherwise specified.

^1^Including Grade III IVH, periventricular hemorrhagic infarction, PVL, and/or severe ventriculomegaly > 15 mm on either side.

Abbreviation: EDI, Early Development Instrument.

Notes: the reported p-values were based on the comparison between 3 groups using Chi-square test or Fisher’s exact test for categorical variables and ANOVA or Kruskal-Wallis test for continuous variables.

**Table A3: NDI characterization of study sample**

| **Areas of neurodevelopmental impairment (NDI)^1^** | **EDI data available (N=50)** |
| --- | --- |
| Bayley-III motor composite score <85 | 23 (47) |
| Bayley-III cognitive composite score <85 | 39 (80) |
| Bayley-III language composite score <85 | 12 (24) |
| ≥2 areas of impairment | 13 (26) |
| Impairment in all areas | 3 (6) |

Data presented as n(%).

^1^“areas” of impairment include neuromotor (cerebral palsy or motor composite score below threshold), neurocognitive (cognitive or language composite scores below threshold) or neurosensory (hearing or vision impairment).

Abbreviations: Bayley-III/IV, Bayley Scales of Infant and Toddler Development, 3^rd^ edition; EDI. Early Development Instrument.
